# Polymorphisms of the cytokine genes *TGFB1* and *IL10* in a mixed-race population with Crohn’s disease

**DOI:** 10.1186/1756-0500-6-387

**Published:** 2013-09-27

**Authors:** Neogelia Pereira Almeida, Genoile Oliveira Santana, Tamara Celi Almeida, Maria Teresita Bendicho, Denise Carneiro Lemaire, Mauricio Cardeal, André Castro Lyra

**Affiliations:** 1 Federal University of Bahia, Salvador, Brazil; 2 Gastroenterology Unit, Roberto Santos General Hospital, Salvador, Bahia, Brazil; 3 Alameda dos Jasmins, n-220, Apt-1902, Cidade Jardim/Candeal, CEP 40296200, Salvador, Bahia, Brazil

**Keywords:** Crohn’s disease, Polymorphism, TGFB1, IL10

## Abstract

**Background:**

Most Crohn’s disease (CD) genes discovered in recent years are associated with biological systems critical to the development of this disease. TGFB1 and IL10 are cytokines with important roles in CD. The aim of this study was to evaluate the association between CD, its clinical features and *TGFB1* and *IL10* gene polymorphisms.

**Methods:**

This case–control study enrolled 91 patients and 91 controls from the state of Bahia, Brazil. Five single nucleotide polymorphisms (SNPs) were studied in the *TGFB1* gene (codon 10 T > C - rs1800470; codon 25 G > C - rs1800471) and *IL10* gene (−1082 A > G - rs1800896; -819 T > C - rs1800871; -592 A > C - rs1800872). An analysis of the genetic polymorphisms was performed using a commercial kit. A comparison of allele frequencies and genotypes was estimated by calculating the odds ratio (OR) with a confidence interval adjusted via the Bonferroni test for a local alpha of 1%. A stratified analysis was applied for gender, race and smoking history. Patients with CD were characterized according to the Montreal classification.

**Results:**

The C allele and CC genotype of the *TGFB1* gene rs1800470 were both significantly associated with CD. The stratified analysis showed no confounding factors for the co-variables of gender, race and smoking history. The *IL10* gene rs1800896 G allele was significantly associated with age at diagnosis of CD, while the T allele of the *IL10* gene rs1800871 was significantly associated with perianal disease. The SNPs rs1800871 and rs1800872 were in 100% linkage disequilibrium.

**Conclusions:**

*TGFB1* gene polymorphisms may be associated with susceptibility to the development of CD, and *IL10* gene polymorphisms appear to influence the CD phenotype in this admixed population.

## Background

Crohn’s disease (CD) pathogenesis is multifactorial, and there appears to be a significant interaction between its genetic, environmental and immunological factors [1,2]. The critical importance of immune regulation in inflammatory bowel disease (IBD) has been recognized, and impairment in immune tolerance to the intestinal microbiota appears to be the primary defect associated with this disease [3,4]. It is interesting to note that TGFB1 and IL10 are the two major immune regulatory cytokines [5-10]. Most CD genes discovered in recent years are associated with biological systems critical to the development of CD, such as innate and adaptive immunity, stress response, autophagy and mucosal barrier [11,12].

TGFB1 is fundamental in maintaining the intestinal epithelial cell homeostasis through its action on modulating T cell activity, dendritic cell function and apoptosis [5-7,10]. The central role of IL10 in the balance of intestinal mucosa immunology is its ability to inhibit the production of proinflammatory cytokines and to regulate the differentiation and proliferation of T and B lymphocytes and NK and antigen presenting cells [5,8-10]. Both TGFB1 and IL10 cytokines play important roles in CD. Animal studies demonstrated the importance of these cytokines in the development of experimental colitis [6,13]. The neutralization of TGFB1 increases Th1 and Th17 responses, and IL10 constrains Th17 cells in patients with CD [6,14]. A defect in TGFB1 production has been observed in patients with CD, and the deregulation of IL10 has been associated with early-onset and severe CD [15,16]. TGFB1 is also involved in fibrosis and stricture formation in CD [17].

Differences in cytokine production have been linked to polymorphisms in gene promoter regions [18-22]. Individual differences in cytokine synthesis could explain the susceptibility to CD and its phenotypic heterogeneity. Studies about the influence of *TGFB1* and *IL10* genetic polymorphisms on the pathogenesis of inflammatory bowel disease (IBD) have been published with controversial results, and most subjects were Caucasians [19,20,23-30].

The *TGFB1* gene has at least two polymorphisms located in the exon 1 region at rs1800470 and rs1800471, which are the most frequently studied polymorphisms in our local population [31,32]. A previous Australian study showed an association between polymorphisms at rs1800471 and CD [33]. The most commonly evaluated polymorphisms in the *IL10* gene are identified in the promoter regions from rs18004896, rs1800871 and rs1800872 [19,20,23,25-32]. These genes are closely related to the expression of IL10 [18,20].

The aim of this study was to analyze the association between CD and the *TGFB1* (codon 10 T > C - rs1800470; codon 25 G > C - rs1800471) and *IL10* (−1082 A > G - rs1800896; -819 T > C - rs1800871; -592 A > C - rs1800872) gene polymorphisms and to examine the association between these polymorphisms and the clinical features of CD, such as age at diagnosis, location, and behavior of the disease in a mixed-race population.

## Methods

This was a case–control study. We evaluated patients from outpatient gastroenterology clinics at the University Hospital Prof. Edgard Santos and at the General Hospital Roberto Santos in Salvador, Bahia. The patients and controls were enrolled between March 2006 and May 2007.

We included patients 18 years or older who had a diagnosis of Crohn’s disease, as established by the clinical, radiological, endoscopic and histopathological features described in the criteria of Lennard-Jones [34]. We excluded first-degree relatives of patients who had been included in the study and patients with indeterminate colitis. The classification for CD was based on the Montreal criteria, which include age at diagnosis, behavior, and location [35]. The ethnic profile was performed using the Krieger criteria [36]. Patients were considered smokers if they had smoked seven or more cigarettes per week for at least one year.

The control group comprised individuals from either outpatient clinics who were 18 years old or older, had been evaluated at the same period of time and had a diagnosis of gastroesophageal reflux disease (GERD) (81 patients) or functional dyspepsia (10 patients). These subjects were required not to have had any neoplastic, infectious or inflammatory diseases, peptic ulcers, diarrhea, hematochezia, fistulas, family history of IBD, or abdominal pain without a specific diagnosis.

To obtain genomic DNA, 10 mL of whole blood was collected from each patient and stored at - 4°C in EDTA tubes until DNA extraction. Genomic DNA was purified using the commercial EZ-DNA kit (Biological Industries, Kibbuts Beit Haemek, Israel) according to the manufacturer’s instructions. The five single nucleotide polymorphisms (SNPs) studied were rs1800470 (codon 10 T > C) and rs1800471 (codon 25 G > C) in the *TGFB1* gene and rs1800896 (−1082 A > G), rs1800871 (−819 T > C), and rs1800872 (−592 A > C) in the *IL10* gene. The SNPs were genotyped with the Cytokine Genotyping Tray Kit (One Lambda, Canoga Park, CA) according to the manufacturer’s instructions. The results were interpreted using maps of the genotyping plates supplied by the manufacturer.

Adherence to the Hardy-Weinberg equilibrium for each polymorphism was tested for both case and control groups using the program GENEPOP [37]. A comparison of allele frequencies and genotypes in the different groups was estimated by calculating the odds ratio (OR) with an adjusted confidence interval (CI) of 99.8% and global α = 0.2% for a local alpha of 1% (Bonferroni adjustment). The co-variables of interest were gender, racial group (evaluated as Whites or African-descendants), and smoking. To analyze the effects of confounding factors and modifiers on the main association, we performed a stratified analysis using estimated ORs with 99% confidence intervals, Mantel-Haenszel adjusted ORs and the Mantel-Haenszel chi-square test. Because no significance was found for a local alpha of 1%, the Bonferroni correction was not considered. Logistic regression was not performed due to insufficient data. To evaluate the association between the polymorphisms and phenotype characteristics of CD, the OR was calculated and Fisher’s exact test was performed, yielding p-values that were adjusted using the Bonferroni method. The global alpha was 0.167%, and the CI was adjusted to 99.83% considering a local alpha of 1%.

Informed written consent was obtained from all subjects, and the study protocol was approved by the Ethics Committee of the Institution (Maternidade Climério de Oliveira - Federal University of Bahia).

## Results

We evaluated 182 subjects, of whom 91 had a definite diagnosis of CD and 91 were controls. The mean age of the patients with CD was 38.0 ± 12.8 years (range 18–75 years, median 37 years), while the control patients had a mean age of 50.3 ± 13.6 years (range 21–88 years, median 50 years). Jewish descent was reported by 3 patients with CD. The distribution of racial groups was similar between the cases and controls. There was a higher frequency of smoking history (current or previous) among the controls. The demographic and clinical characteristics of CD patients and controls are shown in Table 1. When gender, race, and smoking history were compared, there were no statistically significant differences between the cases and controls (data not shown).

**Table 1:** Demographic and clinical characteristics of the CD patients and controls

	**91 Cases (n%)**	**91 Controls (n%)**
• Age		
Mean ± SD	38 ±12.8	50,3 ± 13.6
• Gender		
Female	54 (59.3)	67 (73.6)
Male	37 (40.7)	24 (26.4)
• Race group		
African-descendent	80 (87.9)	82 (90.1)
White	11 (12.1)	09 (09.9)
• Smoking		
No	71 (78.0)	64 (70.3)
Yes	20 (22.0)	27 (29.7)
• Age at diagnosis , n = 91		
A1 ≤ 16 years	12 (13.2)	
A2 17–40 years	55 (60.4)	
A3 > 40 years	24 (26.4)	
• Location , n = 82		
L1 ± L4 Ileum	17 (20.7)	
L2 ± L4 Colon	21 (25.6)	
L3 ± L4 Ileum and colon	43 (52.4)	
L4 Isolated upper gastrointestinal tract	1 (1.3)	
• Behavior, n = 90		
B1 ± P Non-stricturing, non-penetrating	62 (68.9)	
B2 ± P Stricturing	13 (14.4)	
B3 ± P Penetrating	15 (16.7)	
P Perianal disease modifier	40 (44.4)	

n (%) Number of individuals (percent).

All polymorphisms adhered to the Hardy-Weinberg equilibrium in both the case and control groups. The allele frequencies and genotype distribution for *TGFB1* polymorphisms (rs1800470, rs1800471) in the CD patients and controls are shown in Table 2. The C allele of rs1800470 was more frequent in the case group and was associated with CD (p = 0.001, OR = 2.19, 99.8% CI = 1.1-4.42). The CC genotype of rs1800470 was more frequent in the patients with CD compared with the controls (p = 0.001, OR = 4.05, 99.8% CI = 1.31-13.2). These results were statistically significant after a Bonferroni correction. We did not observe any association between *TGFB1* genotypes and the CD phenotype.

**Table 2:** **Allele frequencies and genotype distribution for the*TGFB1*polymorphisms in CD patients and controls**

	**91 Cases n (%)**	**91 Controls n (%)**	***OR***	**[99.8% CI]**	***P***_***B***_	***P value***
**• rs1800470 (codon 10 T > C)**						
T allele	81 (45.5%)	116 (63.7%)	2.19	[1.10- 4.42]	0.005	0.001
C allele	101 (55.5%)	66 (36.3%)				
• Genotype						
TT	22 (24.1%)	39 (42.8%)	Reference			
TC	37 (40.7%)	38 (41.8%)	1.73	[0.66- 4.78]	0.820	0.164
CC	32 (35.2%)	14 (15.4%)	4.05	[1.31- 13.20]	0.005	0.001
**• rs1800471 (codon 25 G > C)**						
G allele	166 (91.2%)	176 (96.7%)	2.83	[0.77-10.89]	0.150	0.030
C allele	16 (8.8%)	6 (3.3%)				
• Genotype						
GG	77 (84.6%)	85 (93.4%)	Reference			
GC	12 (13.2%)	6 (6.6%)	2.21	[0.53-11.08]	0.710	0.142
CC	2 (2.2%)	0 (0%)	¨¨¨¨¨	¨¨¨¨¨¨	........	¨¨¨¨¨¨

n (%) Number of alleles or genotypes (percent).

*P*_*B*_ Bonferroni adjustment.

¨¨¨¨¨¨ Insufficient number.

The allele frequencies and genotypes of the *IL10* polymorphisms (rs1800896, rs1800871 and rs1800872) were similar for both the cases and controls (Table 3). The SNPs rs1800871 and rs1800872 were in 100% linkage disequilibrium. The frequencies of the alleles for these *IL10* SNPs according to the phenotype characteristics of CD are shown in Table 4. The G allele of rs1800896 was more frequent in patients who were 16 years old or younger at diagnosis (p = 0.001, OR = 4.32, 99.83% CI = 1.0022-18.62). An analysis of the association between disease location and *IL10* SNPs was not statistically significant (data not shown). We also found a significantly higher frequency of the T allele of rs1800871 in the *IL10* gene in patients with perianal disease (p < 0.0001, OR = 7.7, 99.83% CI = 2.53-26.90). These results were statistically significant after a Bonferroni correction.

**Table 3:** **Allele frequencies and genotype distribution for the*IL10*polymorphisms in CD patients and controls**

	**91 cases n (%)**	**91 controls n (%)**	***OR***	**[99% CI]**	***P value***
**• rs1800896 (−1082 A > G)**					
Aallele	116 (63.7%)	123 (67.6%)	1.19	[0.66- 2.14]	0.44
G allele	66 (36.3%)	59 (32.4%)			
• Genotype					
AA	41 (45.0%)	40 (44.0%)	Reference		
GA	34 (37.4%)	43 (47.2%)	0.77	[0.32- 1.84]	0.42
GG	16 (17.6%)	8 (8.8%)	1.95	[0.52- 7.45]	0.16
• rs1800871 (−819 T > C)*					
C allele	116 (63.7%)	106 (58.2%)	0.79	[0.45-1.41]	0.28
Tallele	66 (36.3%)	76 (41.8%)			
• Genotype					
CC	36 (39.6%)	28 (30.8%)	Reference		
CT	44 (48.4%)	50 (54.9%)	0.68	[0.28-1.66]	0.24
TT	11 (12.0%)	13 (14.3%)	0.66	[0.17-2.46]	0.38

n(%) Number of alleles or genotypes (percent).

*SNPs rs1800871 and rs1800872 were in 100% linkage disequilibrium.

**Table 4:** **Allele frequencies of the*IL10*polymorphisms according to the Crohn’s disease phenotypes**

			**rs1800896**				rs1800871*		
			**AlleleG**				**Allele T**		
	**N**	**n (%)**	**OR**	***P***_***B***_	***P value***	**n (%)**	**OR**	***P***_***B***_	***P value***
			**[99.83 CI]**				**[99.83 CI]**		
• Age at diagnosis									
A1	12	16 (66.7)	*4.32*	*0.006*	*0.001*	6 (25)	0.54	1.000	0.260
			*[1.0022 – 8.62]*				[0.07 - 2.59]		
A2 + A3	79	50 (31.6)				60 (38)			
• Behavior									
B1	62	42 (33.9)	0.68	1.000	0.316	47 (37.9)	1.4	1.000	0.401
			[0.23 - 2.05]				[0.46 - 4.58]		
B2 + B3	28	24 (42.9)				17 (30.4)			
P	40	30 (37.5)	1.07	1.000	0.877	48 (60)	*7.77*	*< 0.0001*	*< 0.0001*
			[0.38 - 2.96]				*[2.53 - 26.90]*		

A1 ≤ 16 years, A2 17–40 years, A3 > 40 years, B1 Non-stricturing, non-penetrating, B2 Stricturing, B3 Penetrating, P Perianal disease modifier.

N Number of patients, n (%) Number of alleles (percent), P_B_ Bonferroni adjustment.

*SNPs rs1800871 and rs1800872 were in 100% linkage disequilibrium.

The stratified analysis by gender, racial group and smoking history variables in our study showed that none of these factors modified the effect or confounded the association between the studied polymorphisms and CD.

## Discussion

When conducting a case–control genetic association study, the selection of controls is an important issue. This control group should comprise subjects of the same population as that of the cases enrolled [38]. The control group comprised patients treated at the same clinic with similar epidemiological characteristics who were from the same geographical area and had a diagnosis of a clinical condition not associated with the studied polymorphisms. In addition, the controls were prospectively evaluated in the same period of time in which the cases were enrolled. GERD is a motor disorder not genetically determined, and no studies have shown an association between the studied polymorphisms and functional dyspepsia. Both the case and control groups were similar in terms of gender and race. Previous studies from Brazil have shown a genetic admixture from Portuguese, Amerindian and African genetic backgrounds in the population of all studied geographic areas, including the population of Bahia, regardless of the skin color of the individuals [39,40].

Animal studies have shown that a reduction in *TGFB1* affects oral tolerance and results in impaired mucosal immunity [41]. Del Zotto *et al.* performed an interesting study in Italy and observed that low levels of *TGFB1* were present in the intestinal lamina propria of CD patients compared with controls [15]. Our polymorphism analysis of the *TGFB1* cytokine gene showed a positive association between CD and the C allele of SNP rs1800470. However, studies from Europe and North America have failed to show an association between *TGFB1* polymorphisms and CD [23,24,42]. Additionally, the *TGFB1* gene has not been associated with CD in genome-wide association studies (GWAS) [11,12]. The confirmed CD susceptibility loci explained only 23.2% of the disease heritability [11]. These findings could suggest the existence of other genetic variations not captured via GWAS. One possible explanation for the differences between our results and those previously reported is that we investigated African-descent CD patients while the other studies evaluated Caucasian populations. Therefore, the *TGFB1* gene could be directly associated with susceptibility to CD in this population or in a linkage disequilibrium with other genetic markers. This conclusion is biologically plausible, but additional studies are needed to validate these data. The allele frequencies of rs1800470 in *TGFB1* in our CD patients were similar to the frequency of a healthy control group reported by Pereira *et al.*, who evaluated genetic polymorphisms in patients with hepatitis C in the State of Bahia [32]. This result could be because a family history of IBD was not obtained in the hepatitis C study. A patient’s family history of IBD was considered an exclusion criterion in our control group.

TGFB1 is a potent fibrogenic agent that induces the proliferation of fibroblasts and the synthesis of collagen and extracellular matrix [43,44]. Changes in TGFB1 signaling have also been identified in the mucosa overlying strictures in CD [17]. Previous reports have suggested a role for *TGFB1* polymorphisms in stricturing CD and a shorter time to intestinal resection [33]. However, in agreement with our findings, Cantor *et al.* found no association between the *TGFB1* polymorphism and the phenotypic characteristics of CD [23].

The development of spontaneous chronic enterocolitis in genetically modified mice that do not produce the cytokine IL10 has long been known [5,13,16]*.* Many studies have shown no association between several *IL10* polymorphisms and susceptibility to CD [23,25-27,45]. However, studies from Spain, New Zealand, and Mexico showed that these *IL10* polymorphisms were associated with CD susceptibility [19,28,46]. More recently, a meta-analysis showed an association between the SNPs from rs1800896 and CD susceptibility [47]. We have not been able to replicate these findings in our sample.

The *IL10* gene has been identified as a novel CD locus in GWAS studies [11,12]. Experimental studies have shown that a low production of *IL10* is associated with more severe and complex CD [16]. Although usually considered an inhibitory cytokine, *IL10* also stimulates B-cell proliferation [48]. Genetic studies evaluating CD patients demonstrated an association between *IL10* polymorphisms and the severity of disease, including stricturing behavior and pediatric onset [30,49]. We observed a positive association between the G allele of rs1800896 and age at diagnosis of CD. Some studies indicated that the G allele was associated with increased IL10 production [18,20]. The SNPs at rs1800896 appear to dictate early-onset disease, in which the development is likely influenced by genetic rather than environmental factors. The other studied *IL10* SNP (rs1800871) was associated with perianal disease, suggesting that it may be useful in predicting CD behavior. It is possible that the association between specific *IL10* promoter polymorphisms and severe CD may be correlated with the stimulatory effects of *IL10*, but additional functional investigation is necessary to confirm this hypothesis.

CD is considered a frequent disease in Caucasian populations; however, most CD patients in our studied population were not Caucasian. The study of CD in different populations may increase our understanding about the genetics and environmental factors that might interfere with the development CD.

## Conclusion

In conclusion, this study has shown that *TGFB1* polymorphisms may be associated with susceptibility to CD, while *IL10* polymorphisms may influence CD phenotypes. Thus, the polymorphic sites of both genes appear to be particularly important in our population. However, further multicenter studies with larger samples are needed to evaluate the reproducibility of our findings. CD is a disease that greatly impacts patient quality of life, and despite its rising frequency, the pathophysiologic mechanisms of CD are unclear and present a challenge. A better understanding of the various genetic polymorphisms that affect different populations may eventually lead to the better management of CD.

### Availability of supporting data

We have no supporting data to include in an open access repository.

## Competing interests

The authors declare that they have no competing interests.

## Authors’ contributions

ANP and SGO performed the majority of this work; ATC performed the data collection; CM provided the analytical tools; LAC was involved in writing and editing the manuscript; BMTF and LDC performed DNA extraction and genotyping. All authors read and approved the final manuscript.
